# The trichothecene mycotoxin deoxynivalenol facilitates cell‐to‐cell invasion during wheat‐tissue colonization by *Fusarium graminearum*

**DOI:** 10.1111/mpp.13485

**Published:** 2024-06-15

**Authors:** Victoria J. Armer, Martin Urban, Tom Ashfield, Michael J. Deeks, Kim E. Hammond‐Kosack

**Affiliations:** ^1^ Protecting Crops and the Environment, Rothamsted Research Harpenden UK; ^2^ Biosciences University of Exeter Exeter UK; ^3^ Crop Health and Protection (CHAP), Rothamsted Research Harpenden UK

**Keywords:** deoxynivalenol, *Fusarium graminearum*, head scab disease, plasmodesmata, wheat (*Triticum aestivum*)

## Abstract

Fusarium head blight disease on small‐grain cereals is primarily caused by the ascomycete fungal pathogen *Fusarium graminearum*. Infection of floral spike tissues is characterized by the biosynthesis and secretion of potent trichothecene mycotoxins, of which deoxynivalenol (DON) is widely reported due to its negative impacts on grain quality and consumer safety. The *TRI5* gene encodes an essential enzyme in the DON biosynthesis pathway and the single gene deletion mutant, Δ*Tri5*, is widely reported to restrict disease progression to the inoculated spikelet. In this study, we present novel bioimaging evidence revealing that DON facilitates the traversal of the cell wall through plasmodesmata, a process essential for successful colonization of host tissue. Chemical complementation of Δ*Tri5* did not restore macro‐ or microscopic phenotypes, indicating that DON secretion is tightly regulated both spatially and temporally. A comparative qualitative and quantitative morphological cellular analysis revealed infections had no impact on plant cell wall thickness. Immunolabelling of callose at plasmodesmata during infection indicates that DON can increase deposits when applied exogenously but is reduced when *F*. *graminearum* hyphae are present. This study highlights the complexity of the interconnected roles of mycotoxin production, cell wall architecture and plasmodesmata in this highly specialized interaction.

## Graphical abstract

The trichothecene mycotoxin deoxynivalenol (DON) dampens host immune responses and facilitates cell‐to‐cell invasion through plasmodesmatal pit fields in wheat floral tissues.

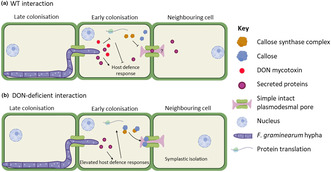

## 1 INTRODUCTION

*Fusarium graminearum* (teleomorph *Gibberella zeae*) is an ascomycete fungal pathogen and the main causative agent of Fusarium head blight (FHB), or scab disease, on wheat. *F*. *graminearum* infects wheat floral tissues at flowering (anthesis), secreting many cell wall‐degrading enzymes (CWDEs), other proteins and metabolites as well as mycotoxins that contaminate the developing grain, rendering it unsuitable for both human and livestock consumption (McMullen et al., 2012). Among these mycotoxins, the sesquiterpenoid type B toxins of the trichothecene class are particularly potent and include deoxynivalenol (DON), nivalenol (NIV), zearalenone (ZEA) and T‐2 toxin (reviewed McCormick et al., 2011), all of which target the ribosome and inhibit protein synthesis (Brown et al., 2004). Trichothecene contamination of grain causes significant economic losses annually (McMullen et al., 1997), destroying wheat crops weeks before harvest and subsequently proliferating during ineffective grain storage/shipment. Epidemics of FHB occur when warm, wet weather coincides with anthesis and are particularly prominent in the mid‐west United States, Asia, Brazil and northern Europe (McMullen et al., 1997; Vaughan et al., 2016). Novel genetic targets are required to help control outbreaks of FHB disease due to the prevalence of resistance to the major class of azole fungicides in global *F*. *graminearum* strains (Fan et al., 2013). Incidences of FHB outbreaks are expected to increase as climate change increases precipitation around wheat harvests (Vaughan et al., 2016). Hence, it is imperative that the infection biology of *F*. *graminearum* is explored further to aid in the development of resistant wheat varieties and precise chemical control, with the overall aim of minimizing FHB‐associated reductions in cereal yields, grain quality and to improve human/animal health.

The infection cycle of FHB commences with the dispersal of conidia (asexual) or ascospores (sexual) by rain droplet‐induced splashes or wind onto wheat plants. During a typical infection of wheat at crop anthesis, germinating spores enter the host floral tissues through natural openings, such as stomata (Pritsch et al., 2007) and cracked open anther sacs, or have been reported to form penetration pegs on the abaxial surface of the palea and lemma tissues of the wheat spikelet (Wanjiru et al., 2002). Host‐tissue colonization continues with the invasive hyphae growing both intercellularly and intracellularly. *F*. *graminearum* has been noted to have a biphasic lifestyle, whereby the advancing infection front is split between macroscopically symptomatic and symptomless phases (Brown et al., 2011). The symptomless phase is hallmarked by apoplastic growth, and the symptomatic by extensive intracellular growth. What initiates this switch is not yet known and is a subject of great interest. During later stages of infection, *F*. *graminearum* secretes CWDEs in abundance (Brown et al., 2012) to facilitate infection by deconstructing wheat cell walls. At the rachis internode, invasive hyphae have been reported to enter vascular elements (Wanjiru et al., 2002) and grow through the remaining wheat spike within the vasculature as well as in the cortical tissue surrounding the vascular bundles (Brown et al., 2010). Furthermore, within the chlorenchyma band of the rachis, *F*. *graminearum* produces perithecia, sexual reproductive structures, completing its lifecycle (Guenther & Trail, 2005). Post‐harvest, *F*. *graminearum* overwinters saprophytically on crop debris or within the soil, thereby infecting subsequent crop cycles. The presence of *F*. *graminearum* in the soil can be the primary cause of seedling blight and root rot in subsequent wheat crops (Parry et al., 1995).

Intracellular growth by *F*. *graminearum* has been previously reported to traverse wheat cell walls though pits or pit fields, where plasmodesmata (PD) are present (Brown et al., 2010; Guenther & Trail, 2005; Jansen et al., 2005). PD are cytoplasmic communication channels that symplastically bridge the cell walls by an appressed endoplasmic reticulum (ER), known as a desmotubule, within a plasma membrane (PM) continuum stabilized by proteins connected to both the ER and PM (Sager & Lee, 2018). PD are instrumental to cellular signalling, allowing for the transport of sugars, ions and small proteins, to name a few. However, plants can adjust the permeability of PD by the deposition of callose, mediated by the action of callose synthases and β‐1,3‐glucanases (Lee & Lu, 2011) at PD junctions. This callose plugging leads to the symplastic isolation of cells that are damaged or under pathogen attack, thereby restricting the movement of secreted pathogen effector proteins, toxins and other metabolites. PD have a major role in host plant defence against viruses, bacteria and fungi (Lee & Lu, 2011). *F*. *graminearum* exploits the plasmodesmatal transit highways by excreting β‐1,3‐glucanases: enzymes that catalyse the breakdown of the 1,3‐*O*‐glycosidic bond between glucose molecules in callose. RNA‐seq analysis of *F*. *graminearum* infection of wheat spikes found that several *Fusarium* β‐1,3‐glucanases are upregulated in the host plant from as early as 6 h post‐infection and peaking at 36–48 h after inoculation (Pritsch et al., 2007).

The trichothecene mycotoxin DON is a well‐reported virulence factor in wheat floral tissues (Cuzick et al., 2008; Jansen et al., 2005; Proctor et al., 1995) and biosynthesis of the toxin requires the *TRI5* gene, encoding the enzyme trichodiene synthase (Hohn et al., 1993). DON is synthesized and then secreted by *F*. *graminearum* hyphae during infection and is a potent ribosomal‐binding translational inhibitor. This broad‐spectrum dampening of induced protein‐dependent defence responses has thus far prevented the elucidation of specific components of host immunity that restrict *F*. *graminearum* in the DON‐deficient interaction. The low molecular weight of trichothecenes and their water‐solubility allow them to rapidly enter cells and target eukaryotic ribosomes. This causes what is known as the ‘ribotoxic stress response’ that can activate, among other processes, mitogen‐activated protein kinases (MAPKs) and apoptosis (reviewed Pestka, 2008). Deletion of *TRI5* eliminates the ability of *F*. *graminearum* to synthesize DON (Proctor et al., 1995), and infection of wheat floral tissues by the single gene deletion mutant (Δ*Tri5*) is restricted to the inoculated spikelet, and results in the production of eye‐shaped lesions on the outer glume (Cuzick et al., 2008; Jansen et al., 2005). Conversely, expression of *TRI5* in wild‐type *F*. *graminearum* is correlated with DON accumulation in planta (Hallen‐Adams et al., 2011). In non‐host pathosystems, such as the model plant species *Arabidopsis thaliana*, infection of floral tissues with the single gene deletion mutant Δ*Tri5* causes a wild‐type disease phenotype, indicating that DON is not a virulence factor in this interaction (Cuzick et al., 2008). Current evidence indicates that the *TRI4* gene, which encodes a multifunctional cytochrome P450 monooxygenase and resides within the main trichothecene mycotoxin biosynthetic cluster (Tokai et al., 2007), is expressed during the *F*. *graminearum–*wheat coleoptile interaction (Qui et al., 2019), but the role of *TRI4* or *TRI5* as a virulence factors in coleoptiles has not yet been reported. The TRI4 protein is required for four consecutive oxygenation steps in trichothecene mycotoxin biosynthesis (Tokai et al., 2007), thus indicating a role for trichothecene mycotoxins during coleoptile infection. Through the use of fluorescent marker reporter strains, the *TRI5* gene has been shown to be induced during infection structure formation on wheat palea (Boenisch & Schafer, 2011). However, the absence of *TRI5* in a *F*. *graminearum* Δ*Tri5‐GFP* strain did not impact the ability of *F*. *graminearum* to form infection cushions during initial time points of infection (Boenisch & Schafer, 2011). The DON mycotoxin naturally occurs as two chemotypes, 15‐ADON and 3‐ADON, and individual *F*. *graminearum* strains secrete either toxin type. The wild‐type (WT) strain used in this study, PH‐1, synthesizes 15‐ADON. Host‐plant resistance to DON is a characteristic of type II FHB resistance, whereby fungal advancement does not proceed beyond the rachis node (reviewed Mesterházy, 1995).

Whilst the macrobiology and some aspects of the cellular biology of the single‐gene deletion mutant Δ*Tri5* have been previously studied, the mode of restriction of Δ*Tri5* remains to be elucidated. Postulations have been made around the role of DON during host‐tissue colonization, specifically relating to the targeting of ribosomes and the subsequent, broad‐spectrum, protein translation inhibition (Pestka, 2010). However, what host defence mechanisms are targeted/specifically affected by DON have not been explored in planta. This study aims to re‐evaluate the infection biology of the Δ*Tri5* strain, and hence the role(s) of DON, during host‐tissue colonization through a combination of molecular and microscopy techniques. Through qualitative and quantitative image analysis of wheat floral tissues during WT and Δ*Tri5* infection, we report that the Δ*Tri5* single gene deletion mutant has an impaired ability to traverse PD. We also find no evidence to support the hypothesis that a general increase in plant cell wall thickening occurs in the absence of DON production, whereby the upregulation of cell wall defences occurs during pathogen attack. From the data gathered, we infer that the secretion of DON during host‐tissue colonization is highly specific both spatially and temporally. This is indicated by the lack of increase in virulence in the Δ*Tri5* mutant when supplied with DON at the point of inoculation in our study. In light of these discoveries, we pose new questions surrounding *F*. *graminearum* infection biology, cell wall colonization and wheat host defence mechanisms.

## 2 RESULTS

The role(s) of DON during *F*. *graminearum* infection of different wheat tissues was addressed through a multifaceted approach. We applied a combination of detailed cell and molecular biology and morphological analyses of floral and coleoptile infections to analyse the effect of DON on hyphae traversing cell walls at PD and the occurrence of the defence response, callose deposition, at PD during infections caused by either the WT strain or the single gene deletion mutant Δ*Tri5 F*. *graminearum* strain.

### 2.1 DON is not required for virulence on wheat coleoptiles and chemical complementation does not restore the WT disease phenotype on wheat spikes

**FIGURE 1:**
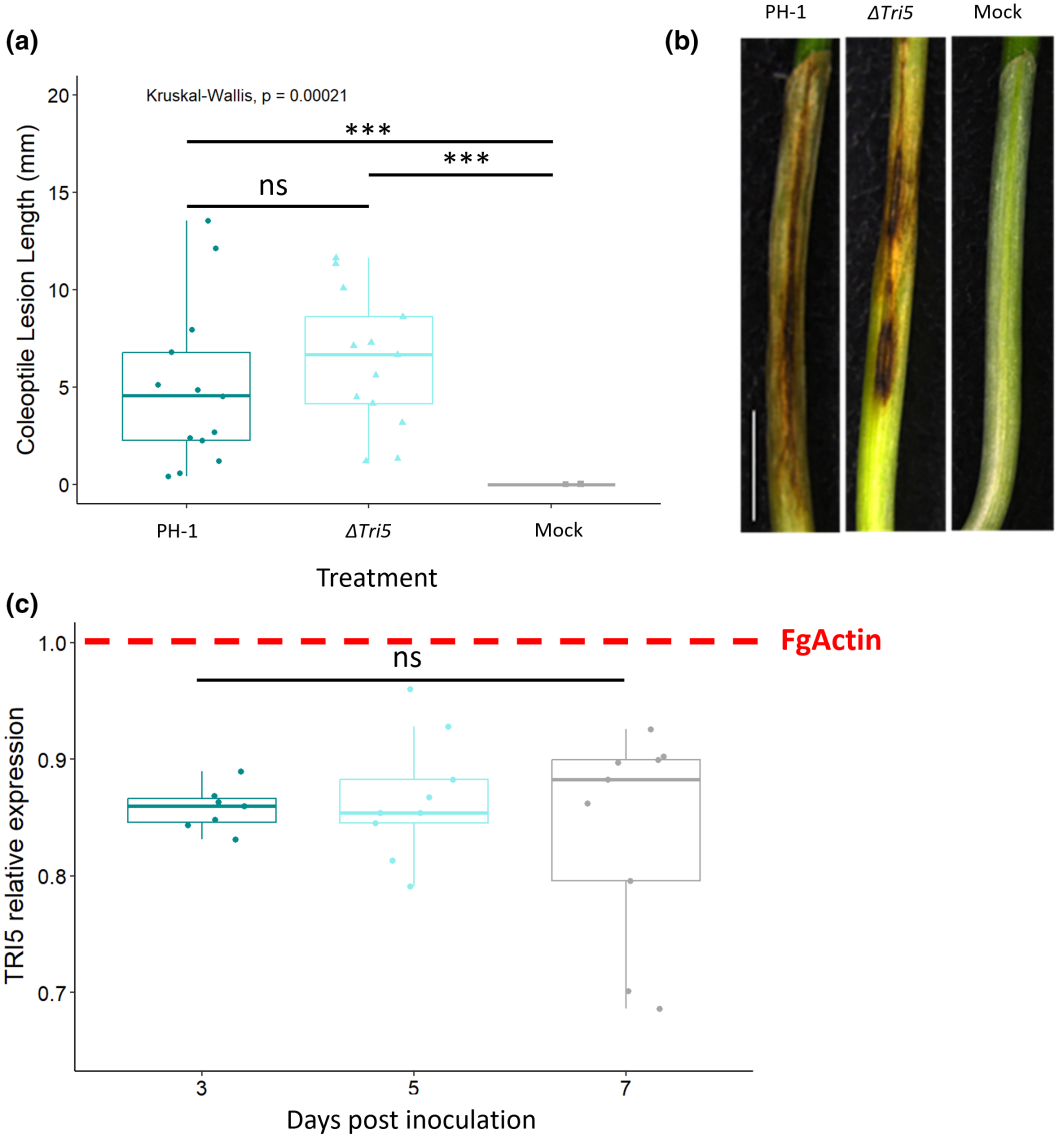
*Fusarium graminearum* disease formation on wheat coleoptiles. (a) Length lesion at 7 days post‐inoculation (dpi) for PH‐1, the Δ*Tri5* mutant and mock inoculations, Kruskal–Wallis ***p < 0.005. (b) Examples of disease lesion phenotypes at 7 dpi for PH‐1, Δ*Tri5* and mock inoculations from rep 2, scale bar = 20 mm and (c) relative expression of *TRI5* measured using reverse transcription‐quantitative PCR at 3, 5 and 7 dpi in wheat coleoptiles, normalized against *FgActin* expression. Analysis of variance *F*(2,22) = 0.421, *p* = 0.662 (ns).

To determine whether DON is or is not required for virulence on wheat coleoptiles under our conditions the fully susceptible cv. Apogee was tested. Inoculation of wheat coleoptiles revealed no differences in lesion length between the WT PH‐1 strain and single gene deletion mutant Δ*Tri5* (Figure 1a,b). However, reverse transcription‐quantitative PCR (RT‐qPCR) analysis showed that the WT strain expressed *TRI5* during coleoptile infection at 3, 5 and 7 days post‐inoculation (dpi), but remains at very low levels (×0.8 *FgActin*) and stationary throughout the infection progression (Figure 1c), indicating that *TRI5* mRNA levels are not temporally altered for this interaction during the early to mid‐time points explored. This finding supports a previous study by Qui et al. (2019), who reported accumulation of transcripts of the *TRI4* gene, also required for trichothecene mycotoxin biosynthesis.

**FIGURE 2:**
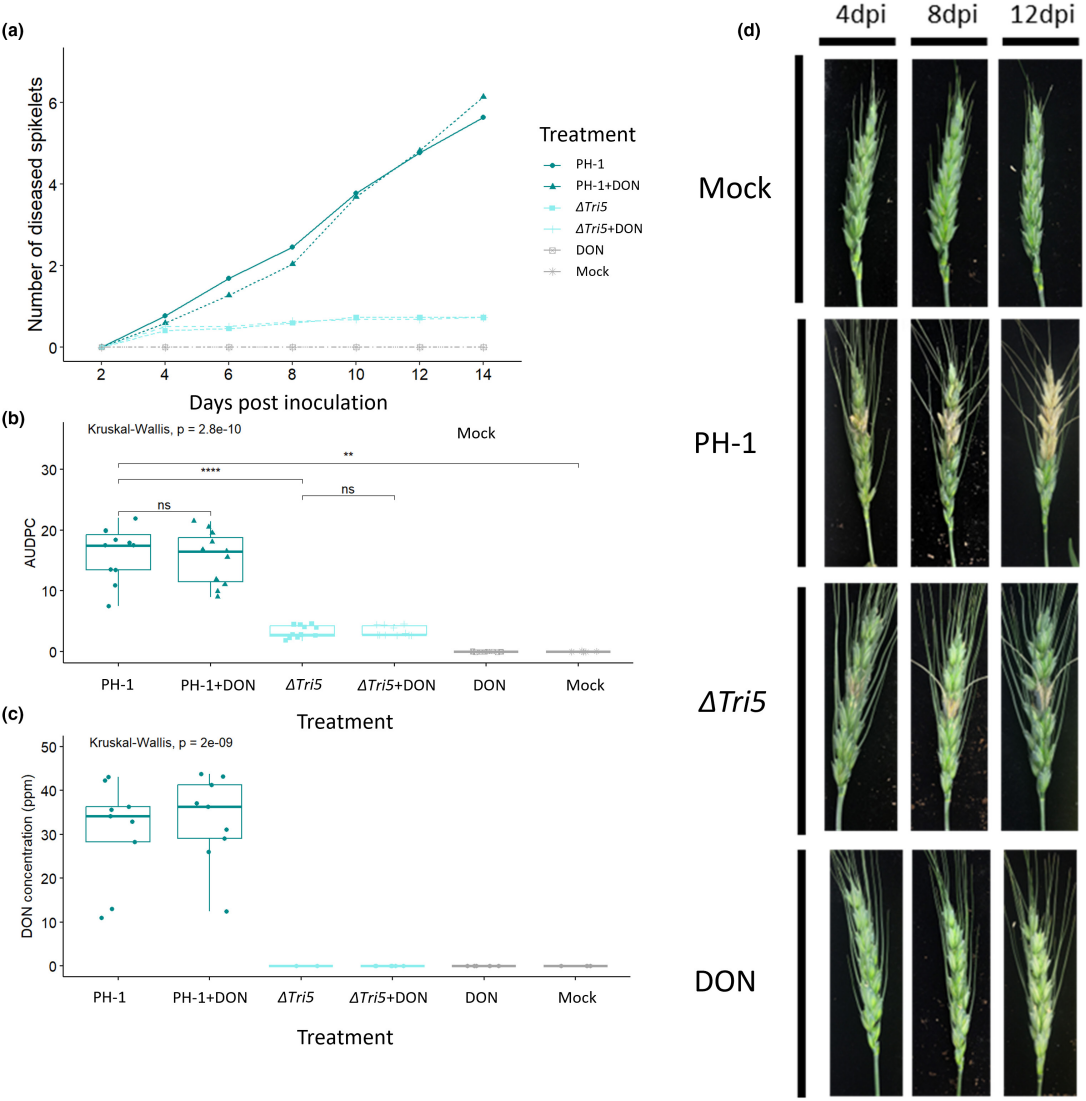
Analysis of whole wheat floral tissues following point inoculations. (a) Tracked visible disease progression at 2 day intervals to 14 days post‐inoculation (dpi) from below the inoculated spikelet. (b) Area under disease progression curve (AUDPC) for disease progression in panel (a) Kruskal–Wallis ***p < 0.005. (c) Deoxynivalenol (DON) concentrations of wheat spikes at 14 dpi, Kruskal–Wallis ***p < 0.005. (d) Representative disease progression images at selected time points of 4, 8 and 12 days.

**FIGURE 3:**
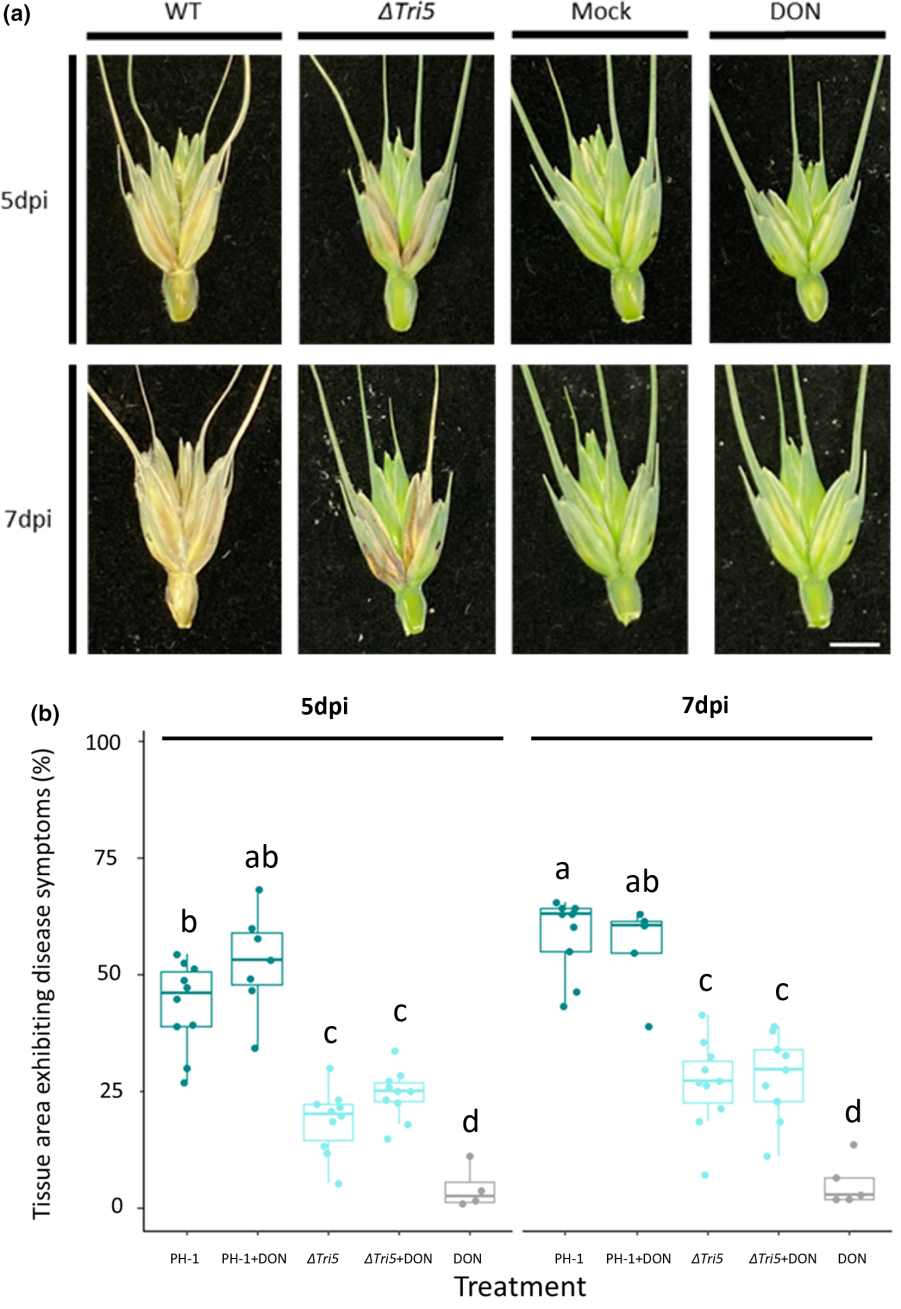
Quantitative spikelet analysis for disease symptom development. (a) Examples of dissected spikelets at 5 and 7 days post‐inoculation (dpi), scale bar = 10 mm. (b) External tissue areas exhibiting disease symptoms at 5 and 7 dpi as determined by Lemnagrid computational software. Analysis of variance, ***p < 0.005, Tukey post hoc denotes group significance.

Next, we asked whether the same host and pathogen genotypes showed different DON dependencies during floral tissue interactions. Disease progression of WT, Δ*Tri5* and DON‐complemented strains were analysed by tracking visible disease symptom development on the outer glume and rachis of inoculated wheat spikes. The single Δ*Tri5* mutant was restricted to the inoculated spikelet in all instances. Chemical complementation of the Δ*Tri5* mutant with DON (35 ppm) applied along with the conidia failed to restore the macroscopic WT spikelet phenotype occurring on the inoculated spikelet or spikelet‐to‐spikelet symptom development. This DON concentration was not detrimental to either spore germination or early spore germling growth (Data S1). Interestingly, co‐inoculation of WT *F*. *graminearum* with DON at the same concentration did not result in any observable advancement of disease symptoms (Figure 2a). Application of DON (35 ppm) alone did not induce any macroscopic disease symptoms and visually equated to the water only (distilled water) mock‐inoculated samples (Figures 2d and 3a). The area under the disease progression curve (AUDPC) analysis revealed that the PH‐1 and PH‐1 + DON supplementation floral infections had significantly greater disease progression than the Δ*Tri5*, Δ*Tri5* + DON, DON only and mock‐inoculated treatments (Kruskal–Wallis, *p* = 2.8e^−10^; Figure 2b). To quantify the levels of DON present in all treatments at the end of disease progression (Day 14), a DON‐ELISA test was carried out to determine the final 15‐ADON concentrations. PH‐1 and PH‐1 + DON samples had an average DON concentration of over 30 ppm, whilst all other treatments had no detectable (<0.5 ppm) DON (Figure 2c). This indicates that the addition of DON to WT inoculum did not stimulate further DON production and confirms that the PH‐1 Δ*Tri5* mutant is impaired in DON biosynthesis. Of note, the lack of detection of DON in the Δ*Tri5* + DON and DON alone samples is probably due to the detoxification of DON by wheat plants to DON‐3‐glucoside, the latter is undetectable by the competitive ELISA kit used in this study. The conjugation of DON to DON‐3‐glucoside, catalysed by a UDP‐glucosyltransferase, in planta is difficult to detect through its increased molecule polarity and is thus known as a ‘masked mycotoxin’ (Berthiller et al., 2005). A visual representation of disease progression occurring in each treatment is shown in Figure 2d.

Cuzick et al. (2008) had previously shown a qualitative difference in the appearance of macroscopic disease symptoms on the glumes between the WT and the Δ*Tri5* mutant. In this study, we have extended this observation and explored the macroscopic and microscopic disease symptoms. Macroscopically, we were able to confirm the Δ*Tri5*‐inoculated spikelets exhibited ‘eye‐shaped’ lesions on the outer surface of the glume by 7 dpi (Figure 3a). These differed from the characteristic fawn brown ‘bleaching’ of the spikelet tissues observed in the WT interaction at 7 dpi (Figure 3a). Chemical complementation of Δ*Tri5* did not restore the WT phenotype nor visibly increase the severity of the WT disease phenotype. To quantify the diseased area, inoculated spikelets were imaged at 5 and 7 dpi and analysed using the Lemnagrid software. The PH‐1 and PH‐1 + DON spikelets had a greater area exhibiting disease symptoms than both the Δ*Tri5* and Δ*Tri5* + DON treatments (Figure 3b). Note that computational restrictions in spikelet parsing from background led to minor, insignificant disease symptoms for DON and mock samples.

### 2.2 The Δ*Tri5* mutant is inhibited in its ability to traverse PD during host‐tissue colonization

**FIGURE 4:**
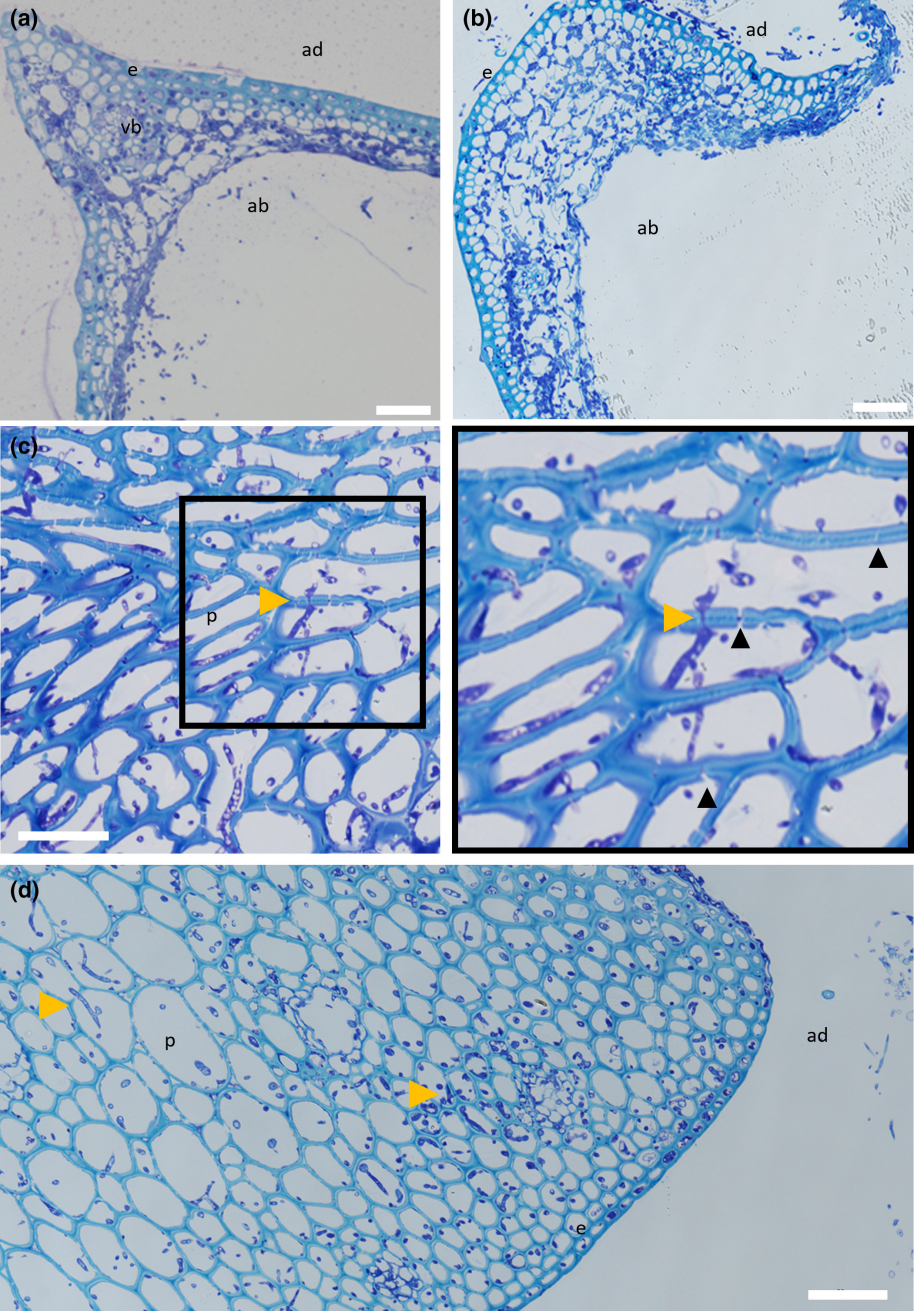
Wild‐type (WT)‐infected wheat floral tissues at 5 and 7 days post‐inoculation (dpi) demonstrating aspects of typical infection. (a) Lemma at 5 dpi infected with WT *Fusarium graminearum* showing widespread hyphal colonization throughout the tissue accompanied by hyphal proliferation protruding from the abaxial layer. (b) A 7 dpi WT‐infected lemma showing further tissue degradation by cell wall‐degrading enzymes and considerable hyphal proliferation. (c) Rachis at 5 dpi infected with WT *F*. *graminearum* showing a number of plasmodesmata (PD) crossings by invasive hyphae, indicated by yellow arrowheads, and extensive cell wall degradation of the mesophyll layer by *F*. *graminearum‐*secreted cell wall‐degrading enzymes. PD can be identified as gaps in the parenchyma layer cell walls, a number of which are indicated by black arrowheads. (d) A 7 dpi WT‐infected rachis demonstrating durability of parenchyma tissue against cell wall‐degrading enzymes at later infection time points. ab = abaxial layer, ad = adaxial layer, e = epidermal layer, p = parenchyma tissue, vb = vascular bundle. Yellow arrowheads indicate PD crossings by invasive *F*. *graminearum* hyphae. Scale bar = 50 μm.

**FIGURE 5:**
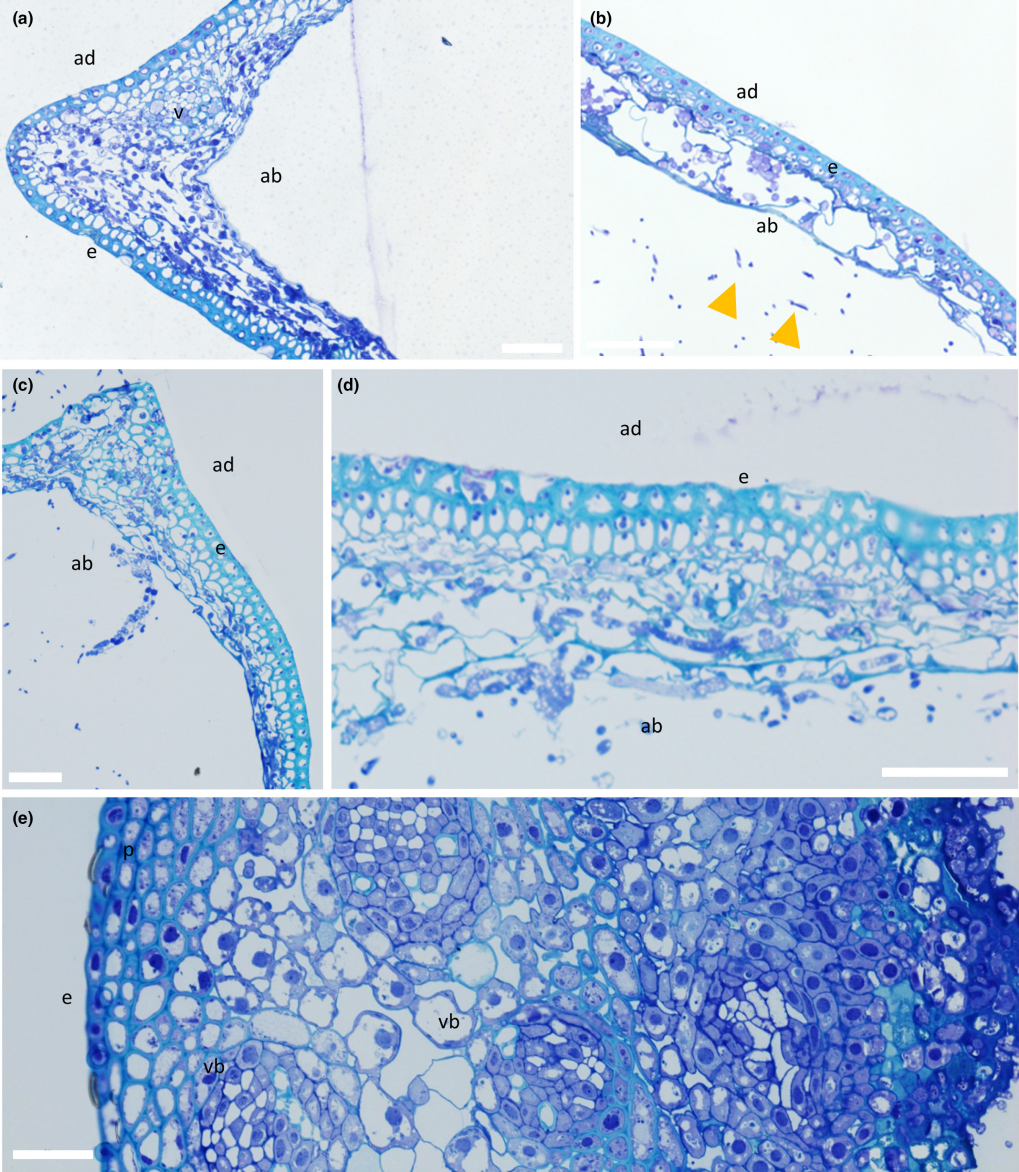
Comparison of Δ*Tri5*‐infected and Δ*Tri5* + deoxynivalenol (DON) infected wheat floral tissues at 5 and 7 days post‐inoculation (dpi) showing the similarities and differences between tissue types in various aspects of a typical infection. (a) Lemma at 7 dpi infected with Δ*Tri5 Fusarium graminearum* with extensive proliferation of invasive hyphae throughout the abaxial layer, but rarely any penetration into the adaxial layer. (b) Palea at 5 dpi infected with Δ*Tri5* and supplemented with 35 ppm DON showing cell wall degradation in the abaxial layer and evidence of external fungal hyphae. Yellow arrows indicate hyphae external to the plant tissue. (c) Palea at 7 dpi infected with Δ*Tri5*, with similar symptoms to the lemma at the earlier 5 dpi time point. (d) Lemma infected with Δ*Tri5* and supplemented with 35 ppm DON at 7 dpi showing cell wall degradation of the abaxial layer. (e) A rachis section at 5 dpi infected with Δ*Tri5* and supplemented with 35 ppm DON. No evidence of hyphae or cell wall degradation throughout the sample. ab = abaxial layer, ad = adaxial layer, e = epidermal layer, p = parenchyma tissue, vb = vascular bundle. No plasmodesmata crossings by invasive *F*. *graminearum* hyphae are evident. Scale bar = 50 μm.

Resin‐embedded samples of the lemma, palea and rachis spikelet components revealed changes in cellular morphology at different points of infection (Figure 4). In the palea and lemma parenchyma tissue layer, the Δ*Tri5* and Δ*Tri5* + DON infected samples exhibited extensive cell wall degradation and colonization by invasive hyphae (Figure 5), similarly to the WT infection. However, in the adaxial layer of the palea and lemma tissues, the hyphae in the Δ*Tri5* and Δ*Tri5* + DON samples rarely penetrated into the thicker‐walled cells (Figure 5a–d). Mirroring the macroscopic lack of symptoms in the rachis, the Δ*Tri5* rachis samples never contained invasive hyphae at either 5 or 7 dpi (Figure 5e). In the PH‐1 and PH‐1 + DON‐infected samples, invasive hyphae proliferated throughout the entirety of the lemma, palea and rachis tissues, causing extensive cell wall degradation (Figure 4). To penetrate the adaxial layer, the PH‐1 hyphae utilized cell wall pits resembling PD pit fields (Figure 4c). In these instances, the hyphae constricted considerably to traverse the cell wall. Traversing of the cell wall through PD pit fields was not observed in Δ*Tri5* and Δ*Tri5* + DON samples at either time point (Figure 5). In general, where hyphae had invaded cells, the cell contents, notably nuclei, chloroplasts and evidence of cytoplasm, were not observed, indicating cell death. In the palea and lemma tissues of the PH‐1 infected samples at 7 dpi, putative evidence of ‘ghost’ hyphae was identified, which are characterized by a lack of cellular contents (Brown et al., 2011) in older infection structures as the infection front advances into the host plant.

To aid elucidation of the role of DON during infection of wheat floral tissues, cell wall thickness from resin‐embedded wheat samples was measured along the adaxial layer of lemma and palea tissues, and in the visibly reinforced regions of rachis tissue, for all treatments. In the adaxial layer of the lemma, palea and rachis tissues, cell wall thickness of resin‐embedded samples imaged by light microscopy was found not to differ between treatments, particularly between those with and without the presence of DON (Data S5). This unanticipated result indicates that cell wall reinforcements are not evident at this level of resolution and are not impacted by the presence of DON. However, it is worth noting that extensive cell wall degradation was present in the abaxial layer of palea and lemma tissues. This microscopic phenotype was not quantified but is most likely caused by the release of CWDEs from *F*. *graminearum* hyphae (Data S5).

**FIGURE 6:**
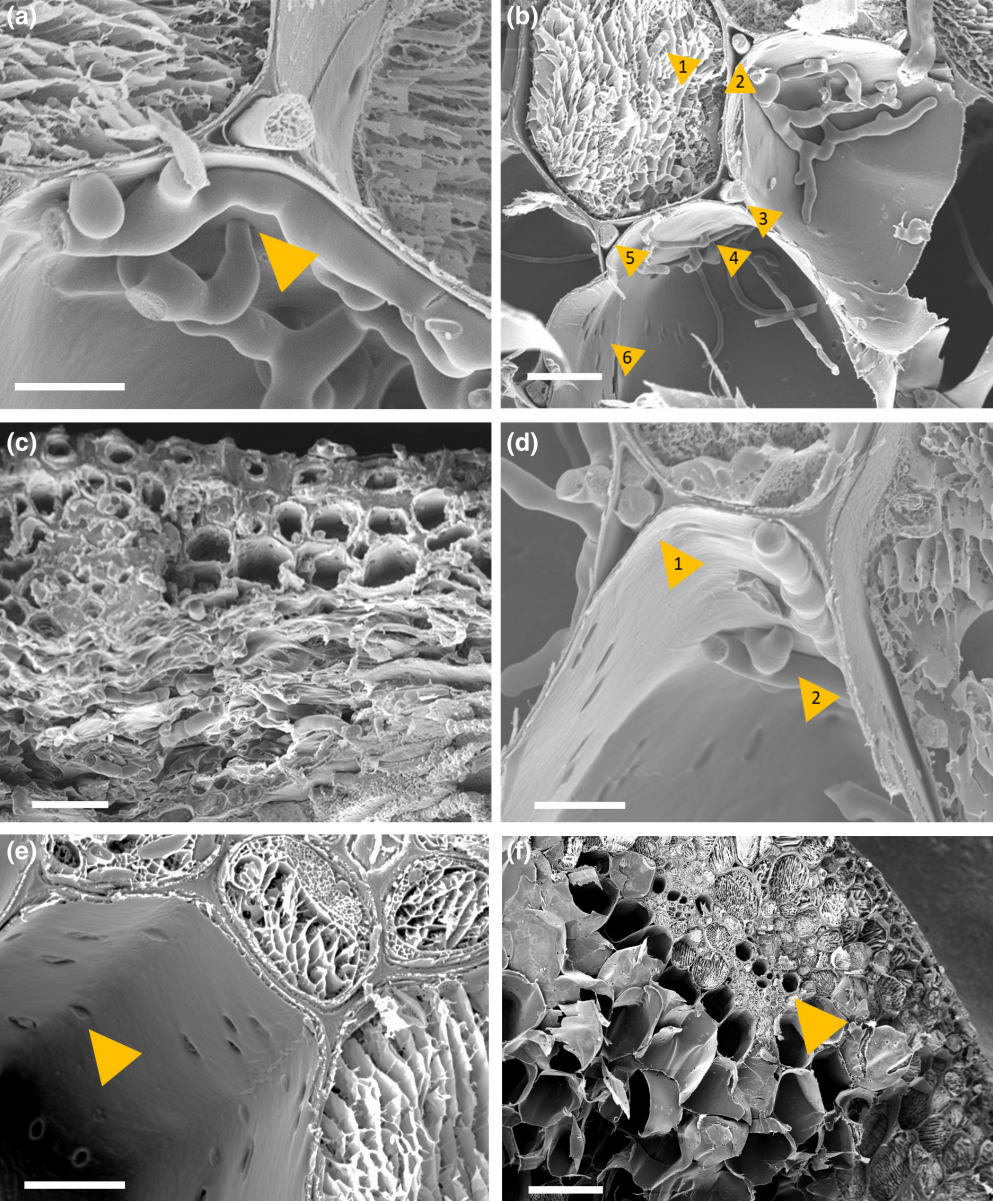
Scanning electron micrographs of *Fusarium graminearum* PH‐1 and Δ*Tri5*‐ wheat floral interactions. (a) A hypha of the wild‐type PH‐1 strain appears to cross through the cell wall at 5 days post‐inoculation (dpi) in rachis tissue. Scale bar = 10 μm. (b) Wild‐type PH‐1 infecting rachis tissue at 5 dpi, the numbered yellow arrowhead indicates point of interest. 1. Intracellular growth in a cell where cytoplasm is still present; 2., 3., and 5. Apoplastic growth between cells, 4. Potential crossing of the cell wall by a hypha through a plasmodesma and 6. ‘Holes’ in the cell wall that are potential sites of plasmodesmata. Scale bar = 20 μm. (c) Δ*Tri5*‐infected lemma tissue at 5 dpi demonstrating extensive hyphal colonization and cell wall degradation of the parenchyma tissue layer (bottom), but minimal infection in the thicker‐walled adaxial layer (top), scale bar = 20 μm. (d) Wild‐type PH‐1 infection of the rachis at 5 dpi, 1. Growth of two hyphae through the same apoplastic space in parallel to hyphae growing intracellularly in neighbouring cells to the left and right. 2. Hypha appear to constrict to traverse the cell wall. (e) Mock‐inoculated rachis tissue, yellow arrow indicates pores in cell wall. (f) Mock‐inoculated rachis tissue showing lack of cellular contents in the central regions of the tissue. Yellow arrow indicates vascular bundle. (a)–(e) Scale bar = 10 μm, (f) scale bar = 100 μm.

In order to gain a thorough understanding of infection, a scanning electron microscopy (SEM) analysis was used. SEM micrographs of rachis post‐spikelet inoculation with WT PH‐1 at 5 dpi revealed several notable interactions, including intracellular growth through cells still containing cytoplasm, apoplastic growth between cells, hyphal constriction and cell wall traversing and gaps in rachis cell walls (Figure 6a,b,d). Micrographs of Δ*Tri5‐*infected lemma tissue at 5 dpi confirmed the resin analysis, whereby extensive cell wall degradation was observed in the parenchyma tissue layer (Figure 6c).

### 2.3 Immunolabelling of callose during infection reveals reduced deposits in the WT infection and phloroglucinol staining indicates lignin‐based defence response(s)

**FIGURE 7:**
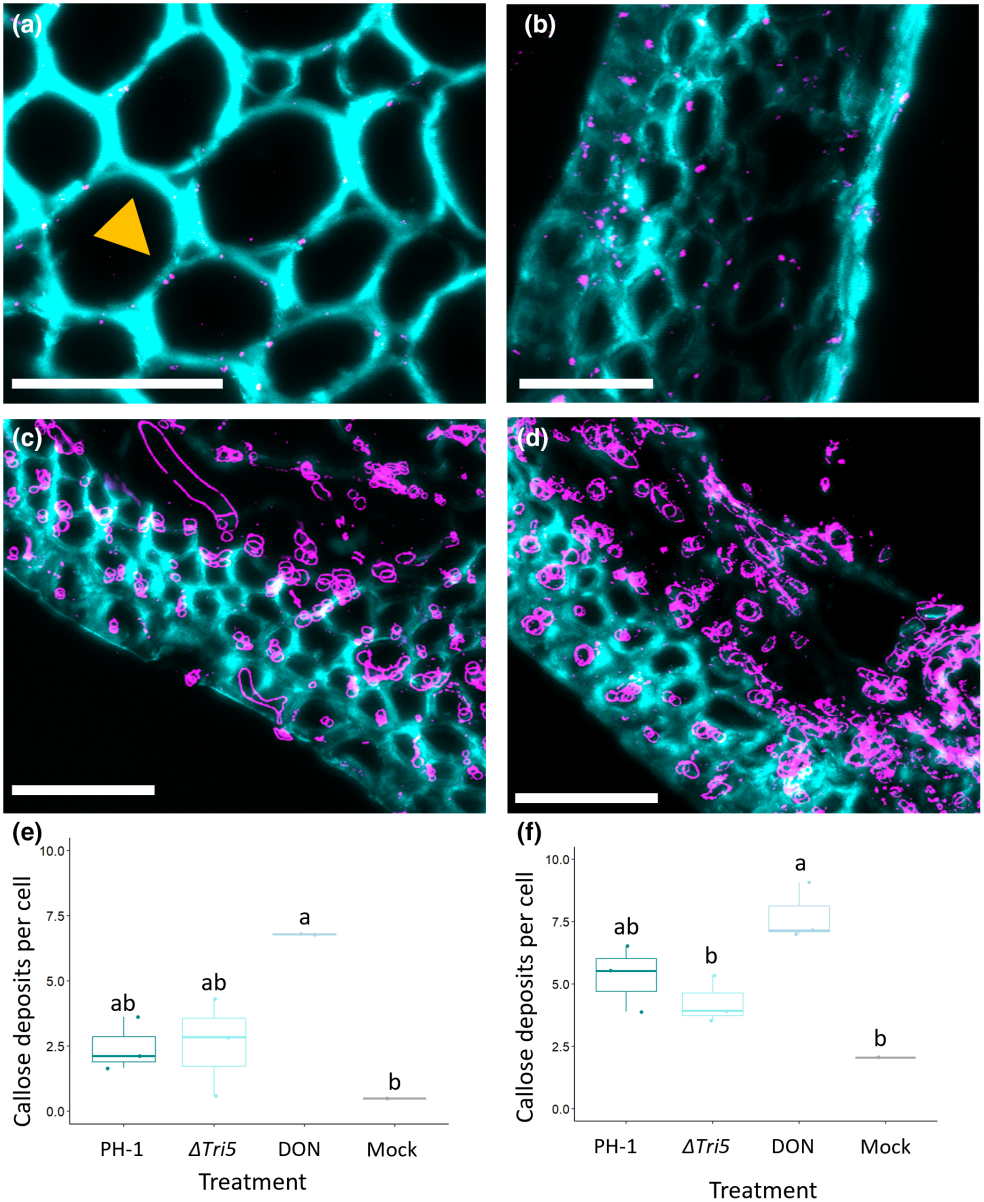
Immunofluorescence detection of callose in rachis and lemma tissues. Magnified region of interest of the *Fusarium graminearum–*wheat interaction demonstrating callose deposits at plasmodesmata. (a) Control rachis, yellow arrow indicates callose deposit at break in cell wall staining, (b) dexoynivalenol (DON)‐inoculated lemma tissue, (c) *F. graminearum* PH‐1 infected lemma at 5 days post‐inoculation (dpi), (d) Δ*Tri5*‐infected lemma at 5 dpi. Sections were imaged by confocal microscopy with excitation‐emission spectra for Alexa Fluor‐488 at 488 nm, 510–530 nm and 405 nm, 450–475 nm for Calcofluor white. Callose deposits are labelled in magenta with wheat cell walls in cyan. Scale bars = 50 μm. In panels (c) and (d) the *Fusarium* hyphae also react positively to the antibody due to β‐1,3‐glucans in the fungal cell wall and are labelled in magenta. (e) Quantification of the number of immunolabelled callose deposits, averaged across number of cells in the sample area, in lemma tissues at 5 dpi infected with wild‐type (PH‐1), ΔTri5, DON and mock control, analysis of variance (ANOVA), *p < 0.05, and (f) rachis tissues at 5 dpi infected with PH‐1, Δ*Tri5*, DON and mock controls, ANOVA, *p < 0.05. Letters indicate significance differences between groups from Tukey post hoc analysis following one‐way ANOVA.

Resin sections of PH‐1, Δ*Tri5* and mock‐inoculated wheat floral tissues were analysed for the presence of callose at junctions in the cell wall (Figure 7). Immunolabelling for the presence of callose confirmed the material of pit structures was consistent with PD. Imaging revealed that in both WT (PH‐1) and DON‐deficient (Δ*Tri5*) *F*. *graminearum*‐inoculated spikes there was an increased frequency of instances where callose was deposited at PD junctions compared to mock‐inoculated controls (Figure 7). However, the DON‐only inoculated samples exhibited a marked increase in callose in both lemma and rachis tissues, indicating that callose deposition had been induced in a manner consistent with a basal immune response to symplastically isolate cells after the detection of the DON toxin.

Spikelets of wheat inoculated with WT, PH‐1 and Δ*Tri5* were sampled at 5 dpi for analysis of the lignin response. This investigation was prompted by the presence of localized eye‐shaped lesions in the Δ*Tri5‐*infected samples. Darker staining by the phloroglucinol indicates a higher lignin content, which was found to be most notable in the Δ*Tri5‐*infected lemma tissue (Data S8). This was surprising, as the lesions were present on the glume. Whilst this was not quantified, the WT PH‐1 and mock‐inoculated controls were visually comparable, indicating that WT *F*. *graminearum* may have a role in dampening pathogen‐induced lignin upregulations, possibly through the action of DON. This proposes the hypotheses that in the absence of trichothecene mycotoxins, wheat is able to upregulate lignin defence pathways.

## 3 DISCUSSION

This study has re‐examined and extended knowledge on the restricted host tissue colonization phenotype previously reported in wheat spikes for the non‐DON‐producing Δ*Tri5* single gene deletion mutant of *F*. *graminearum*. The study was catalysed by the lack of published cellular information available on how DON, produced and secreted by the advancing *F*. *graminearum* hyphae, actually facilitates the extraordinary effective and speedy disease progression consistently observed in the spikes of susceptible wheat cultivars. DON has long been classified as a key virulence factor in the *F*. *graminearum–*wheat interaction (Hohn et al., 1993; Jansen et al., 2005) and facilitates the host‐tissue colonization of the rachis and thus is essential for successful internal spikelet‐to‐spikelet growth of hyphae through the entire floral spike. However, prior to this study, the morphological and cellular responses underlying this macroscopically well‐documented phenomenon had not been explored. In this study our two primary aims were (a) to identify the morphological differences in the hyphal infection routes between the WT and Δ*Tri5* strains during wheat floral infections compared to coleoptile infections, and (b) to focus on the multifaceted role(s) of the cell wall and its constituent components as well as the pit fields during hyphal colonization due to their potential to delay, minimize or cease fungal progression through the numerous internal complexities that the wheat spike architecture presents to the *Fusarium* hyphae.

As described above, our experimentation confirmed that the Δ*Tri5* mutant could sufficiently colonize the lemma and palea tissues but not the rachis (Cuzick et al., 2008; Jansen et al., 2005). Similarly, our results concurred with those of Jansen et al. (2005) that the DON‐deficient *F*. *graminearum* strain could not grow beyond the rachis node due to the presence of inherently thicker cell walls in this tissue. However, our quantitative comparative analysis of the WT and DON‐deficient interactions revealed no differences between cell wall thickness at two time points, or with the control mock‐inoculated tissues, indicating that cell walls do not increase in thickness per se as part of a locally occurring defence response. Upon further microscopic analysis in the current study, we observed that the DON‐deficient Δ*Tri5* mutant could not enter wheat cells with inherently thicker cell walls because the hyphae could not pass through pit fields containing PD. This phenomenon was frequently observed in both the cortical and sclerenchyma cell layers. As a result, the Δ*Tri5* hyphae accumulated within and between the neighbouring thinner‐walled parenchyma cells. In the absence of DON, potentially other so far uncharacterized secreted proteinaceous effectors fail to correctly manipulate these potential gateways into the neighbouring wheat cells. The analysis of resin sections revealed that cell walls within the adaxial layer of lemma and palea tissues were not thicker in infected samples. Although this rules out additional cell wall reinforcements, these findings do not eliminate cell wall compositional changes. Our results indicate that lignin content increases in the lemma tissue, which strengthens the tissue and hence emphasizes the role of PD as cell wall portals in host‐tissue colonization. We hypothesize that DON, through its intracellular target of the ribosomes, inhibits local protein‐translation‐based defence responses, whereas symplastic isolation of neighbouring cells by the deposition of callose at PD is a largely post‐translationally regulated process induced within the generic plant defence response (Wu et al., 2018). Our SEM inquiry of the infected tissues indicates that PD, when used by the advancing hyphal front, are potentially ‘dead portals’ that lack the desmotubule symplastic bridge between neighbouring cells, but this needs to be confirmed through further analysis by transmission electron microscopy. This would also confirm whether callose deposits are eliminated prior to or coincident with hyphal constriction and traversing of the cell wall. Although again a static analysis method, transmission electron microscopy can be used to explore whether desmotubule connections, and callose deposits, are consistently present or absent at the point of hyphal traverse. Collectively, these data suggest that the broad‐spectrum consequences of DON targeting could prevent the synthesis and action of key defence enzymes at PD. This could be explored by a combined comparative proteomics, phosphoproteomics and RNA‐seq analysis of the WT and Δ*Tri5*‐infections to elucidate the wheat defence responses occurring at the advancing *Fusarium* hyphal front that are reduced and/or eliminated by the presence of DON.

The deposition of callose at the PD junction by callose synthases has been demonstrated to be induced by various biotic stress‐inducing pathogens (Wu et al., 2018). The role of callose differs with cellular location: callose polymers are a structural component of papillae in various cereal species that form below appressoria produced by fungal pathogens such as the powdery mildew *Blumeria graminis* f. sp. *hordei*, whereby elevated callose deposition in highly localized papillae in epidermal cells results in resistance to fungal infection (Ellinger et al., 2013). In vascular tissue, callose can be deposited to restrict vascular advancements by wilt pathogens, including by *Fusarium* and *Verticillium* species (reviewed in Kashyap et al., 2021). To investigate the potential of DON impacting upon PD occlusion following our discovery of the impeded traversal of PD by the Δ*Tri5* strain, we immunolabelled callose in resin‐embedded sections of wheat floral tissues. We found that DON strongly induced callose depositions, and callose deposition was also moderately increased in WT‐ and Δ*Tri5*‐infected lemma and palea tissues. This indicates that callose deposition is increased as a defence response when DON or *Fusarium* hyphae are present. However, in the WT infection, we observed a frequency of callose depositions similar to the non‐DON producing Δ*Tri5* strain indicating an interruption or targeted degradation of callose occlusions by *F*. *graminearum* invasive hyphae. We note, however, that the chemical fixation process employed for this analysis could affect the integrity of callose deposits. The secretion of glycoside hydrolase (GH) proteins that break down β‐1,3‐glucans such as callose has not been explored with respect to the *F. graminearum–*wheat interaction, although GH12 family proteins that break down xyloglucan in plant cell walls appear to be implicated in virulence (Wang et al., 2022). In the Δ*Tri5* infections, in the absence of DON other hyphal components and/or secreted molecules may be responsible for the modest callose deposition at the PD junction.

Intracellular colonization through the rachis node and beyond in the rachis internode possibly requires DON and is therefore required for the second intracellular phase of the biphasic lifestyle described for *F*. *graminearum*, where extracellular apoplastic growth characterizes the initial ‘stealth’ phase of infection (Brown et al., 2010). If this is the case, then lacking the ability to traverse PD would restrict direct acquisition of nutrients from host cells by the fungal hyphae. The *TRI* biosynthetic gene cluster required for DON biosynthesis is transcriptionally activated early during wheat spike infection, peaking between 72 and 120 h post‐inoculation (Chen et al., 2019; Evans et al., 2000), when infection is largely restricted to the palea, lemma and glume tissues. Trichothecene biosynthesis is regulated by two transcription factors, TRI6 and TRI10, within the biosynthetic pathway. Of note, DON is not required for full virulence of the developing wheat kernel seed coat (Jansen et al., 2005). In addition to our finding in coleoptiles, Ilgen et al. (2009) identified that trichothecene biosynthesis pathway induction was potentially tissue specific and somewhat restricted to the developing grain kernel and rachis node, and suggested that ‘kernel tissue perception’ by the *Fusarium* hyphae induces the biosynthesis of trichothecene mycotoxins. This suggestion concurs with Zhang et al. (2012) who reported that the trichothecene biosynthesis genes were not induced during *F*. *graminearum* infection of wheat coleoptiles, whereas Qui et al. (2019) reported the expression of *TRI4* in wheat coleoptiles. We found *TRI5* expression to be detectable at low levels in the wheat coleoptiles, remaining around 0.8× the expression levels of *FgActin*. This is lower than the *TRI5* expression level reported by Brown et al. (2011), who reported a level over 3× that of *FgActin* in wheat spikes.

Gardiner, Osbourne, et al. (2009) presented evidence that, in addition to their previous reports that exogenous application of amines, such as agmatine, in vitro induces *TRI5* expression (Gardiner, Kazan, & Manners, 2009), low pH further accelerates expression of the *TRI* gene cluster. Other inducers of the DON biosynthetic pathway genes include carbon, nitrogen and light (Gardiner, Osbourne, et al., 2009). These factors could explain the low and stationary levels of *TRI5* gene expression throughout infection of wheat coleoptiles, which have been noted to be particularly acidic plant tissue due to the optimum activity of expansion proteins around pH 4 (Gao et al., 2008). Other fungal pathogens that are reported to use PD during infection of cereals include *Magnaporthe orzyae* and *M*. *oryzae* pathotype *triticum*, which respectively cause rice blast and wheat blast diseases on the floral panicles and floral spikes (Fernandez & Orth, 2018; Sakulkoo et al., 2018). Although *M. oryzae* does not synthesize trichothecene mycotoxins, the invading hyphae secrete another potent general protein translation inhibitor, namely tenuazonic acid (Wilson & Talbot, 2009). The effect of this mycotoxin on PD traversing and virulence in *Magnaporthe* spp. has not yet been reported.

*F*. *graminearum* progression into the rachis and through sequential rachis nodes and internodes allows for the successful completion of the disease infection cycle in wheat crops. Typically, perithecia form from the chlorenchyma band of the rachis following prolific hyphal colonization of this highly specialized photosynthetic tissue layer within the wheat spike (Guenther & Trail, 2005). Hence, interruption of WT disease progression prior to this crucial point in the primarily monocyclic infection cycle is of great interest for reducing full virulence of FHB and in particular in reducing the abundance of air‐dispersed ascospores. Interestingly, infection of barley spikelets with WT *F*. *graminearum* is solely restricted to the inoculated spikelet, similarly to Δ*Tri5* infection of wheat (Jansen et al., 2005). How this occurs has not yet been explored, but we hypothesize that a lack of traversing of PD by hyphae may have a role to play in barley rachis node tissue.

**FIGURE 8:**
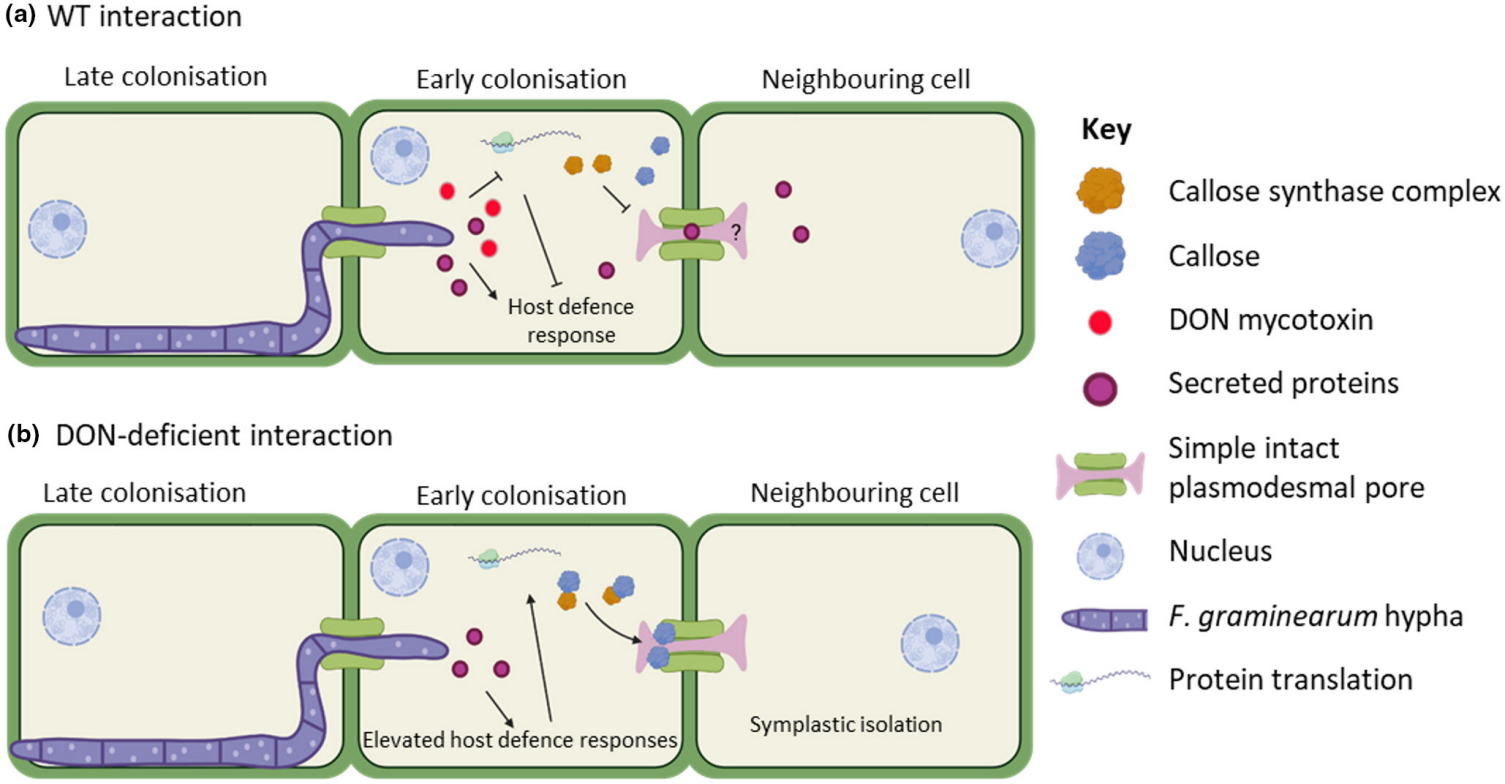
Proposed working model for the role of deoxynivalenol (DON) in the *Fusarium graminearum*–wheat interaction. (a) In the wild‐type (WT) interaction, DON interferes with the wheat host defence response by inhibiting protein translation and reducing the ability of the host to deposit callose at plasmodesmata (PD) to restrict further hyphal infection. It is currently unknown how long the desmotubule remains functional or in place. (b) In the absence of DON, *F. graminearum*‐secreted proteins are detected by the host and trigger host defence responses, including the symplastic isolation of neighbouring cells by the deposition of callose at PD.

Overall, our study indicates that PD are the key to successful host‐tissue colonization by *F*. *graminearum* and that DON, directly or indirectly, facilitates this interaction. We anticipate that the results of this study are considered in future working disease models of the *F*. *graminearum–*wheat interaction and suggest these incorporate a greater emphasis on tissue and cell wall architecture and composition when considering host susceptibility to fungal pathogens. To this end, we have proposed a new working model (Figure 8) that summarizes our findings around the presence of DON during wheat infection and the impact on callose deposition at PD.

## 4 EXPERIMENTAL PROCEDURES

### 4.1 Fungal growth

The *F. graminearum* reference strain PH‐1 (NCBI: txid229533) and the DON‐deficient single gene deletion mutant Δ*Tri5*, within the PH‐1 parental background (Cuzick et al., 2008), were used in this study. Conidia for glycerol stocks were prepared by culturing on synthetic nutrient‐poor agar (SNA) plates containing 0.1% KH_2_PO_4_, 0.1% KNO_3_, 0.1% MgSO_4_·7H_2_O, 0.05% KCl, 0.02% glucose, 0.02% sucrose and 2% agar. Plates were left to grow for 8 days at room temperature (RT) with constant illumination under near‐UV light (Philips TLD 36W/08). TB3 liquid medium (0.3% yeast extract, 0.3% Bacto peptone and 20% sucrose) was added to plates to stimulate spore production and left for a further 2 days. Conidia were harvested and stored in 15% glycerol at −80°C in 2 mL cryotubes (Thermo Fisher Scientific). Conidial suspensions in water to be used for inoculations were prepared by spreading conidia from glycerol stocks onto potato dextrose agar (PDA; Sigma Aldrich) plates. The plates were then incubated at RT for 2 days. After incubation, conidia were harvested with distilled water. Spore concentrations were measured with the aid of a haemocytometer (Hausser Bright‐line). Experiments were conducted under APHA plant licence number 101948/198285/6.

### 4.2 Plant growth

The susceptible dwarf spring wheat (*Triticum aestivum*) cultivar Apogee was used for all wheat experiments, sourced from the National Small Grains Collection, USDA‐ARS, Aberdeen, Idaho, United States. Seeds were sown in Rothamsted Prescription Mix (RPM) soil (Petersfield Growing Mediums) in P15 pots (approx. volume 7 cm^3^) and grown in controlled environment facilities at HSE category 2 (Fitotron; Weiss Gallenkamp), 16 h light:8 h dark cycle at 22°C and 18°C, respectively, 70% relative humidity and illumination at 2200 μmol m^−2^ s^−1^.

### 4.3 Coleoptile inoculations

For coleoptile inoculations, Apogee grains were left for 2 days at 5°C in water for imbibition before being placed individually onto cotton wool plugs in a 24‐well tissue culture plate (VWR) and left to germinate for 3 days under high humidity conditions (<90% relative humidity) under normal wheat growth conditions. At 3 days after sowing, approximately 5 mm from the tip of each coleoptile was cut to encourage infection. Inoculations occurred through the placement of a cut pipette tip with a filter paper insert soaked with 5 × 10^5^ spores/mL solution, with distilled water used as a negative control. The coleoptiles were left in the dark for 3 days to aid infection, after which inoculation tips were subsequently removed, and coleoptiles were left to grow under normal growth conditions for a further 4 days (Darino et al., 2024). Disease phenotypes on the coleoptiles were assessed at 7 days post‐inoculation by imaging lesions on an M205 FA stereomicroscope (Leica Microsystems). Each experimental replicate contained five biological samples for each treatment (three mock‐inoculated) and the experiment was repeated three times.

### 4.4 Floral inoculations

At mid‐anthesis, wheat plants were inoculated with 5 × 10^5^ spores/mL water conidial suspension of PH‐1 or Δ*Tri5*, conidial suspension supplemented with DON, DON alone or water (distilled water) control. DON supplementation of inoculum was 35 ppm (Sigma‐Aldrich). As described in Lemmens et al. (2005), a 5‐μL droplet was placed between the palea and the lemma on each side of the seventh true spikelet from the base. Inoculated plants were placed in a high (<90%) humidity for the first 72 h of infection, with the first 24 h in darkness. After 72 h plants were returned to normal growth conditions.

### 4.5 Determination of DON concentration for exogenous application

To determine the concentration of DON to use for the chemical complementation experiments, a number of experiments were undertaken. These scoped for a concentration that was not detrimental to both *F*. *graminearum* spore germination and growth (Data S1), as well as sufficient to cause physiological stress to plant‐tissue in the plant model *Arabidopsis thaliana* (Data S2). *A. thaliana* was grown as detailed in Armer et al. (2024). Concentrations up to 350 ppm were not detrimental to fungal proliferation in media and as low as 10 ppm demonstrated considerable reduction in growth in *A. thaliana* seedlings. The concentration of 35 ppm (118 μM) was determined as a result of these experiments.

### 4.6 Disease progression

As above, Apogee at mid‐anthesis was inoculated and disease progression was assessed by counting spikelets showing visible symptoms every 2 days after inoculation until 14 dpi. Area under disease progression curve (AUDPC) (Van der Plank, 1963) values were calculated using the ‘agricolae’ package (v. 1.4.0; de Mendiburu & Yaseen, 2020) in R (v. 4.0.2). Statistical significance was determined by Kruskal–Wallis one‐way analysis of variance through the R package ‘ggplot2’ (v. 3.4.0; Wickham, 2016).

### 4.7 Red green blue colour classification for disease assessment of dissected spikelets

To quantify disease progression on wheat spikelets at 5 and 7 dpi, colour (RGB) spikelets were imaged (iPhone 6s; Apple) on both sides with consistent illumination. Diseased area was quantified using a curated program on the LemnaTec Lemnagrid software (CHAP). Diseased area was classified by pixel colour segmentation after application of filters to threshold from the background, identify misclassified pixels and fill in gaps. Area attributed to anthers were omitted from further analysis. The relative area attributed to each classification was then calculated in a custom R script and all samples were normalized to the mean value of ‘diseased’ of the mock treatment due to background parsing error.

### 4.8 Bioimaging

Inoculated spikelets were dissected from the wheat spikes for internal observations of infected floral tissues. Spikelets were fixed for 24 h in a solution of 4% paraformaldehyde, 2.5% glutaraldehyde and 0.05 M Sorensen's phosphate buffer (NaH_2_PO_4_:Na_2_HPO_4_·7H_2_O, pH 7.2), in the presence of Tween 20 (Sigma‐Aldrich) and subject to a light vacuum for 20 s to ensure tissue infiltration. Fixed spikelets were washed three times with 0.05 M Sorensen's phosphate buffer and subsequently underwent an ethanol dehydration protocol at 10% ethanol increments, up to 100% ethanol. Spikelets were dissected into component tissues and embedded with LR White resin (TAAB) at increasing resin ratios (1:4, 2:3, 3:2, 4:1), followed by polymerization in the presence of nitrogen at 60°C for 16 h. Ultrathin 1 μm resin sections were cut from resin blocks using a microtome (Reichert‐Jung; Ultracut), placed onto polysine microscope slides (Agar Scientific) and stained with 0.1% (wt/vol) Toluidine Blue O in 0.1 M Sorensen's phosphate buffer. Every 10th section was collected for a total of 10 sections per embedded block to fully explore floral tissues and mounted with Permount (Fisher Scientific) prior to imaging on an Axioimager 512 (Zeiss) at 20× magnification under brightfield illumination. The experiment was repeated three times, with a total of five biological replicates for each treatment, with two mock samples per batch. In total, 111 resin blocks were explored across a 100 μm in the centre of the sample, with sections cut every 10 μm. Image analysis was conducted in Fiji for ImageJ (v. 2.3.0) and statistical analysis was conducted in R (v. 4.0.2).

For SEM exploration of floral tissues, spikelets at 5 dpi were excised and coated in 50:50 OCT compound (Sakura FineTek) with colloidal graphite (TAAB). SEM analysis was conducted on rachis tissue infected with the WT reference strain PH‐1 at 5 dpi. Sample preparation occurred in a Quorum cryo low‐pressure system before imaging on a JEOL LV6360 SEM at 5 kV with software v. 6.04.

For the analysis of cell wall thickness in WT, WT + DON, Δ*Tri5*, Δ*Tri5* + DON, DON and mock treatments at 5 and 7 dpi, fixed resin sections were used. For each treatment, five biological replicates were used and 10 non‐adjacent cell wall measurements across the sample made using Fiji for ImageJ (v. 2.3.0), with the average used for statistical analysis.

Callose immunolabelling of resin‐embedded sectioned material was conducted according to Amsbury and Benitez‐Alfonso (2022). Briefly, callose was localized by anti‐β‐1,3‐glucan antibodies (Biosupplies) and secondarily conjugated with rabbit anti‐mouse Alexa Fluor‐488. Wheat cell walls were counterstained with Calcofluor white. Sections were imaged by confocal microscopy on a Leica SP8 confocal microscope, with excitation‐emission spectra for Alexa Fluor‐488 at 488 nm, 510–530 nm and 405 nm, 450–475 nm for Calcofluor white. Image analysis for the quantification of callose deposits per cell was conducted in Fiji (ImageJ) using maximum projections of *Z* stacks and channels converted to binary masks. The number of cells in the sample area was calculated using the cell counter tool and callose deposits were counted by the number of discrete Alexa Fluor‐488 fluorescent objects between the size of 2 to 12 pixel units to eliminate cross‐reactivity with β‐1,3‐glucans in the fungal cell walls. The number of callose deposits were averaged across the number of complete cells (all cell walls visible) in the sample area. The same image analysis parameters were set to all images, including mock treatments. Callose deposits were quantified in the lemma and rachis tissues only, with three biological replicates for each treatment (PH‐1, Δ*Tri5*, DON, Mock). Further examples are present in Data S6 and image analysis methodology is demonstrated in Data S7.

### 4.9 DON quantification

To determine if the presence of DON in the WT strain inoculum stimulated further DON production, if administered DON could be detected in wheat spike tissues at the end of disease progression (14 dpi), and the absence of DON in the Δ*Tri5* mutant interaction a competitive ELISA for 15‐ADON was employed. Whole wheat spikes were used for this quantification in the case the DON was trafficked or secreted beyond the inoculated spikelet due to its high water solubility. Whole wheat spikes after 14 days of disease progression were ground to a fine powder in the presence of liquid nitrogen and 1 g of each sample was resuspended in 5 mL distilled water, vortexed until dissolved, incubated in a 30°C water bath for 30 min and centrifuged for 15 min at full speed (13,100 *g*). The supernatant was removed and analysed using the Beacon Analytical Systems Inc. Deoxynivalenol (DON) Plate Kit according to kit instructions. The A_450_ values were measured on a Varioskan microplate reader (Thermo Scientific). Three technical replicates of each biological replicate (a single wheat spike) were conducted, and the experiment was repeated three times.

### 4.10 Expression of the mycotoxin biosynthesis gene *TRI5* during coleoptile infection

The trichodiene synthase gene *TRI5* was used as a proxy for the relative expression of the trichothecene biosynthesis pathway during coleoptile infection. Total RNA was extracted from whole coleoptiles at 3, 5 and 7 dpi using a total RNA extraction kit (NEB) and following the kit instructions. First‐strand cDNA was synthesized using RevertAid First Strand cDNA synthesis kit (ThermoFisher Scientific) as per kit instructions and using random hexamer primers provided. *TRI5* expression was then assessed by qPCR with melt curve using the primers in Data S3, with SYBR Green as the reporter, ROX as passive reference and NFQ‐MGB as the quencher. The qPCR with melt curve was conducted in technical and biological triplicate on a QuantStudio 6 Pro and results analysed on the complementary Design & Analysis Software v. 2.6.0 (ThermoFisher Scientific). The experiment was conducted three times. Quantification of *TRI5* expression was normalized against the housekeeping gene *FgActin* (primers in Data S3) for each sample, also included within the qPCR run in triplicate. At least one primer in a primer pair for qPCR targets was designed to span an exon‐exon junction to avoid annealing to residual DNA.

### 4.11 Phloroglucinol staining for presence of lignin

A 3% phloroglucinol (Sigma Aldrich)‐HCl solution (Weisner stain) was prepared fresh in accordance with methods by Mitra and Loque (2014). Inoculated wheat spikelets were sampled at 5 dpi and cleared in 100% ethanol for 4 days before going through a rehydration series (75%, 50%, 25% and 0% ethanol) at 1 h per stage. Cleared spikelets were bathed in Weisner stain for 1 h, or until staining of the tissues became evidently saturated. Spikelets were then imaged (OM‐D E‐M10; Olympus) under constant illumination and, subsequently, dissected tissues were imaged individually.

### 4.12 Formation of perithecia in vitro

Carrot agar was prepared using the method outlined by Cavinder et al. (2012) and supplemented with DON at 35 ppm (wt/vol) to test for the ability of the WT strain, and the DON trichothecene‐deficient deletion mutant, Δ*Tri5*, to develop perithecia in vitro, for lifecycle completion viability (Data S4). Ability of perithecia to discharge ascospores in the presence of DON was assessed using the same method as described in Cavinder et al. (2012).

### 4.13 Statistical analysis

Scripts were written in R (v. 4.0.2) for each experimental analysis. Unless otherwise stated, analysis of variance followed by Tukey post hoc test was conducted for parametric datasets and Kruskal–Wallis for nonparametric datasets. The significance threshold was set to *α* = 0.05 in all cases.

## CONFLICT OF INTEREST STATEMENT

The authors declare no financial or non‐financial competing interests.

## Supporting information


Data S1.

Data S2.

Data S3.

Data S4.

Data S5.

Data S6.

Data S7.

Data S8.

## Data Availability

The research data supporting this publication are provided within this paper. Requests for materials relating to this paper should be made to Kim Hammond‐Kosack (kim.hammond-kosack@rothamsted.ac.uk) at Rothamsted Research.
