# Supplementary material for: The trichothecene mycotoxin deoxynivalenol facilitates cell‐to‐cell invasion during wheat‐tissue colonization by Fusarium graminearum
Source: Mol Plant Pathol. 2024 Jun 15;25(6):e13485. doi: 10.1111/mpp.13485 (PMC11178975; doi:10.1111/mpp.13485)
Supplement: Supplementary file 1 — Data S1. [file MPP-25-e13485-s001.docx]

## S1 Determination of the DON concentration to be used in point inoculations with *F. graminearum* conidia

To determine the suitable concentration of DON for inoculum supplementation, a pilot experiment was conducted to demonstrate a lack of toxicity to *F. graminearum* at various concentrations ranging from 0.5 - 10 times concentrations used *in planta*. The 1.5ml microcentrifuge tubes containing 1ml YPD (Formedium Ltd, UK) were inoculated to a concentration of 5x10^4 spores/ml and incubated at 28°C for 24 hours with rotation at 180 rpm. Hyphal growth was measured at 6, 12, 18 and 24 hours after inoculation by light microscopy and analysed on Fiji image processing software (v. 2.3.0) (Schindelin et al., 2012). The concentration selected for the *in planta* experiment (35ppm DON) was considered to be non-detrimental to spore germination or early spore germling growth.
