# Supplementary material for: The trichothecene mycotoxin deoxynivalenol facilitates cell‐to‐cell invasion during wheat‐tissue colonization by Fusarium graminearum
Source: Mol Plant Pathol. 2024 Jun 15;25(6):e13485. doi: 10.1111/mpp.13485 (PMC11178975; doi:10.1111/mpp.13485)
Supplement: Supplementary file 2 — Data S2. [file MPP-25-e13485-s007.docx]

**S2 Toxicity of DON on plant tissue**

Due to the inability to easily detect deoxynivalenol (DON; chemotype 15-ADON) in plant tissues once glycosylation has occurred, and the lack of macroscopic necrosis observed in wheat spikelets post-inoculation with water containing 35 ppm DON, we set out to confirm the potent toxicity of the DON chemistry (sourced from Sigma-Aldrich, USA) on plant tissues. We utilised an assay described by Shin et al. (2012) and DON concentrations of 0mg/l, 10mg/l (10ppm, 33μM) and 20mg/l (20ppm, 67μM) to produce comparable phenotypes on *Arabidopsis thaliana* (ecotype Col-0; NASC, UK) during germination and early growth. Briefly, Col-0 seeds were surface sterilised and germinated on the surface of ½ MS 1% (w/v) agar plates (as described in Armer et al., 2024), with each plate supplemented with a different DON concentration or no DON. We found significant reductions in both the speed of germination and subsequent seedling growth with increasing concentrations of DON, with a notable absence of root proliferation when DON was present, demonstrating its toxicity to plants.
