# Supplementary material for: The trichothecene mycotoxin deoxynivalenol facilitates cell‐to‐cell invasion during wheat‐tissue colonization by Fusarium graminearum
Source: Mol Plant Pathol. 2024 Jun 15;25(6):e13485. doi: 10.1111/mpp.13485 (PMC11178975; doi:10.1111/mpp.13485)
Supplement: Supplementary file 3 — Data S3. [file MPP-25-e13485-s006.docx]

## S3 Primers used in this study (5’-3’)

| **Target** | **Sense (LP)** | **Anti-sense (RP)** | **Use** |
| --- | --- | --- | --- |
| FgActin | ATGGTGTCACTCACGTTGTCC | CAGTGGTGGAGAAGGTGTAACC | For RNA expression in wheat coleoptiles, determined by qPCR of cDNA. |
| TRI5 | TCCGTAGCACTATGGACTTTTT | TAGGATGGGCTTCTGAGCCT |  |
