# Supplementary material for: The trichothecene mycotoxin deoxynivalenol facilitates cell‐to‐cell invasion during wheat‐tissue colonization by Fusarium graminearum
Source: Mol Plant Pathol. 2024 Jun 15;25(6):e13485. doi: 10.1111/mpp.13485 (PMC11178975; doi:10.1111/mpp.13485)
Supplement: Supplementary file 4 — Data S4. [file MPP-25-e13485-s003.docx]

## S4 Perithecia formation *in vitro* does not require DON

In natural floral infections in the wheat fields of North America, the typical scab disease symptoms are caused by perithecial development during the later stages of crop maturation (Guenther and Trail, 2004). To determine whether the *ΔTri5* mutant could successfully sexually reproduce, and thereby determine if DON is required for this process, perithecia were induced *in vitro* using a highly reproducible method. The *ΔTri5* mutant was able to produce abundant perithecia that were macroscopically indistinguishable in size to those produced by WT PH-1. Furthermore, to test the viability of formed perithecia, successful ascospore discharge from perithecia was observed by light microscopy for both the WT and *ΔTri5 F. graminearum* strains.
