# Supplementary material for: The trichothecene mycotoxin deoxynivalenol facilitates cell‐to‐cell invasion during wheat‐tissue colonization by Fusarium graminearum
Source: Mol Plant Pathol. 2024 Jun 15;25(6):e13485. doi: 10.1111/mpp.13485 (PMC11178975; doi:10.1111/mpp.13485)
Supplement: Supplementary file 5 — Data S5. [file MPP-25-e13485-s008.docx]

**S5 Cell wall thickness of adaxial cell layer in resin samples.**

*
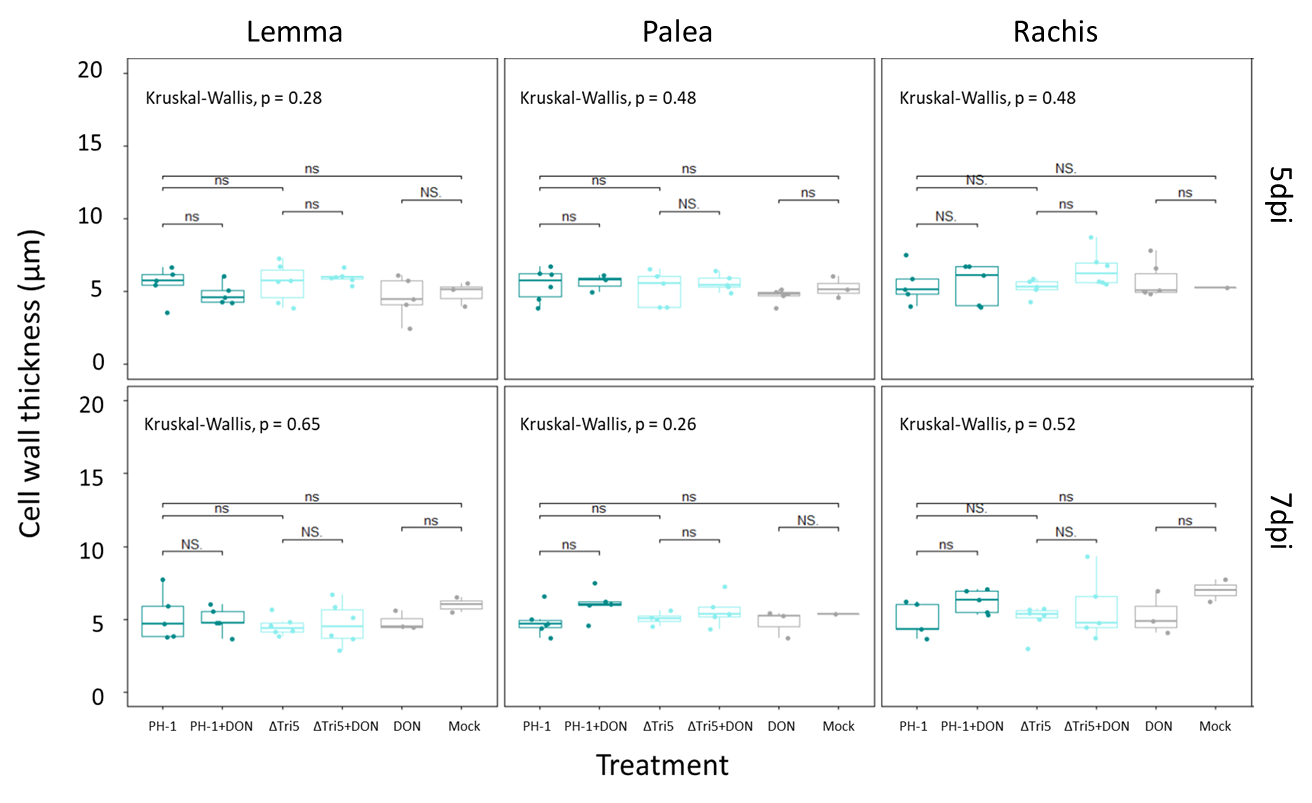
*

Wheat spikelet tissues of palea, lemma and rachis at 5 and 7dpi time points were analysed, with an average of 10 measurements from a representative resin image of each biological replicate analysed. No significance was determined by Kruskal-Wallis.
