# Supplementary material for: The trichothecene mycotoxin deoxynivalenol facilitates cell‐to‐cell invasion during wheat‐tissue colonization by Fusarium graminearum
Source: Mol Plant Pathol. 2024 Jun 15;25(6):e13485. doi: 10.1111/mpp.13485 (PMC11178975; doi:10.1111/mpp.13485)
Supplement: Supplementary file 6 — Data S6. [file MPP-25-e13485-s004.docx]

## S6 Immunofluorescence detection of callose in sectioned floral tissues


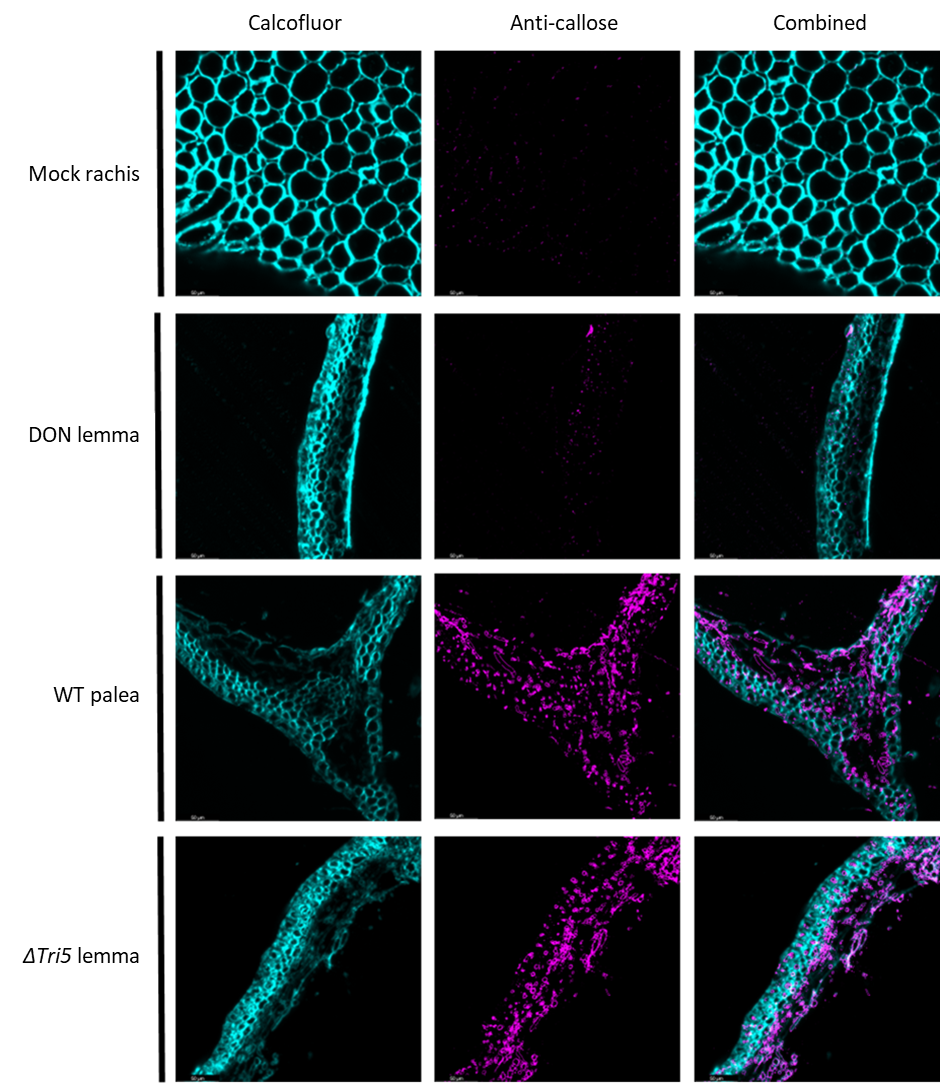


**Immunofluorescence detection of callose in sectioned floral tissues.** Wheat floral tissues from palea, lemma and rachis embedded in LR white resin and sectioned to 1µm then immuno-labelled with anti-callose antibodies and secondarily conjugated with AlexaFluor-488 (magenta). Wheat cell walls are counterstained with calcofluor white (cyan). Selected images of Control, PH-1 (WT) , DON, and *ΔTri5*-infected wheat floral tissues at 5dpi demonstrating typical observations of each interaction.
