# Supplementary material for: The trichothecene mycotoxin deoxynivalenol facilitates cell‐to‐cell invasion during wheat‐tissue colonization by Fusarium graminearum
Source: Mol Plant Pathol. 2024 Jun 15;25(6):e13485. doi: 10.1111/mpp.13485 (PMC11178975; doi:10.1111/mpp.13485)
Supplement: Supplementary file 7 — Data S7. [file MPP-25-e13485-s002.docx]

**S7 Quantification of immuno-labelled callose deposits in wheat spikelet resin sections**


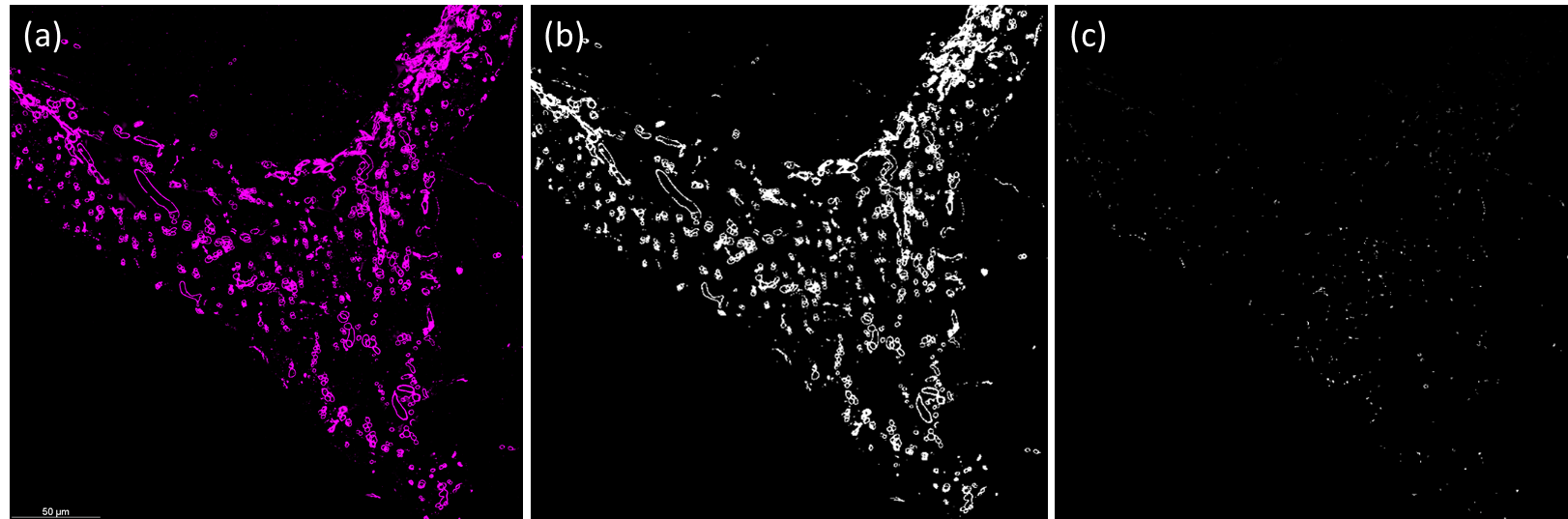


**Example methodology for the quantification of immunofluorescence detection of callose in sectioned floral tissues.** Wheat palea tissue infected with the wildtype PH-1 strain of *F. graminearum* at 5dpi and immuno-labelled for callose, with the secondary antibody conjugated to the fluorophore Alexa Fluor-488. (a) the RGB channel for the emission spectra 510nm- 530nm to detect callose (magenta). β-1,3-glucans in the fungal cell wall have cross-reactivity with the anti-callose antibody. (b) RGB images are converted into binary masks for measurement of particles. (c) Using the Analyse Particles tool in Fiji, particles are counted between 2-19 pixel units in size. This reduces counts due to noise (1 pixel in size) but eliminates pixels attributed to β-1,3-glucans in the fungal cell wall.
