# Supplementary material for: The trichothecene mycotoxin deoxynivalenol facilitates cell‐to‐cell invasion during wheat‐tissue colonization by Fusarium graminearum
Source: Mol Plant Pathol. 2024 Jun 15;25(6):e13485. doi: 10.1111/mpp.13485 (PMC11178975; doi:10.1111/mpp.13485)
Supplement: Supplementary file 8 — Data S8. [file MPP-25-e13485-s005.docx]

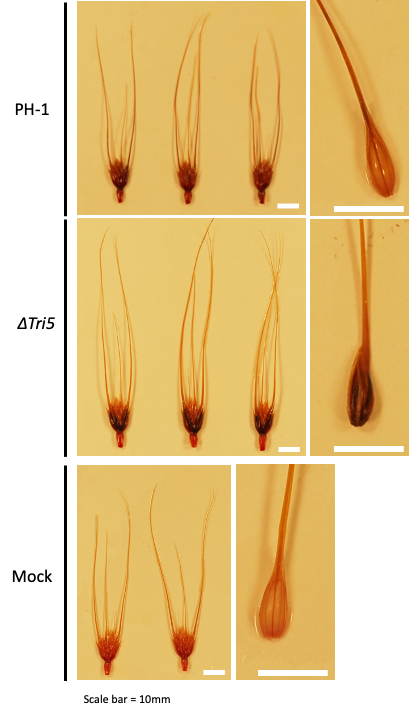
**S8 Phloroglucinol staining of infected spikelets for the detection of lignin.**

Darker staining of the tissues indicates a greater quantity of lignin. (a) PH-1 - infected spikelet, (b) *ΔTri5-*infected spikelet, (c) Mock-inoculated spikelet. Spikelet component tissues: Lemma demonstrated an increase in phloroglucinol staining component, shown to the left of each treatment, indicating an increase in lignin content. N.B. Point inoculations occur between the lemma and palea tissues. All spikelets were collected at 5dpi and are of the wheat cv. Apogee. Scale bar = 10mm.
